# A new high-quality genome assembly and annotation for the threatened Florida Scrub-Jay (*Aphelocoma coerulescens*)

**DOI:** 10.1093/g3journal/jkae232

**Published:** 2024-09-27

**Authors:** Faye G Romero, Felix E G Beaudry, Eyvind Hovmand Warner, Tram N Nguyen, John W Fitzpatrick, Nancy Chen

**Affiliations:** Department of Biology, University of Rochester, Rochester, NY 14620, USA; Department of Biology, University of Rochester, Rochester, NY 14620, USA; Clinical Translation, Ontario Institute for Cancer Research, Toronto, ON M5G 0A3, Canada; Department of Biology, University of Rochester, Rochester, NY 14620, USA; Department of Ecology & Evolutionary Biology, Cornell University, Ithaca, NY 14853, USA; Department of Ecology & Evolutionary Biology, Cornell University, Ithaca, NY 14853, USA; Cornell Lab of Ornithology, Cornell University, Ithaca, NY 14850, USA; Department of Biology, University of Rochester, Rochester, NY 14620, USA

**Keywords:** Florida Scrub-Jay, Corvidae, de novo genome assembly, linkage map, pedigree

## Abstract

The Florida Scrub-Jay (*Aphelocoma coerulescens*), a federally Threatened, cooperatively breeding bird, is an emerging model system in evolutionary biology and ecology. Extensive individual-based monitoring and genetic sampling for decades has yielded a wealth of data, allowing for the detailed study of social behavior, demography, and population genetics of this natural population. Here, we report a linkage map and a chromosome-level genome assembly and annotation for a female Florida Scrub-Jay made with long-read sequencing technology, chromatin conformation data, and the linkage map. We constructed a linkage map comprising 4,468 SNPs that had 34 linkage groups and a total sex-averaged autosomal genetic map length of 2446.78 cM. The new genome assembly is 1.33 Gb in length, consisting of 33 complete or near-complete autosomes and the sex chromosomes (ZW). This highly contiguous assembly has an NG50 of 68 Mb and a Benchmarking Universal Single-Copy Orthologs completeness score of 97.1% with respect to the *Aves* database. The annotated gene set has a Benchmarking Universal Single-Copy Orthologs transcriptome completeness score of 95.5% and 17,964 identified protein-coding genes, 92.5% of which have associated functional annotations. This new, high-quality genome assembly and linkage map of the Florida Scrub-Jay provides valuable tools for future research into the evolutionary dynamics of small, natural populations of conservation concern.

## Teaser

Romero *et al*. present a new high-quality genome assembly and annotation for the Florida Scrub-Jay (*Aphelocoma coerulescens*), a Federally Threatened bird species. In comparison to other genome assemblies of this species, the authors’ assembly is the first one generated using long-read sequencing technology and from a female individual. They also constructed the first linkage map for this species. The authors’ genome assembly is highly contiguous and is of similar quality to other bird genome assemblies.

## Introduction

**Fig. 1.**
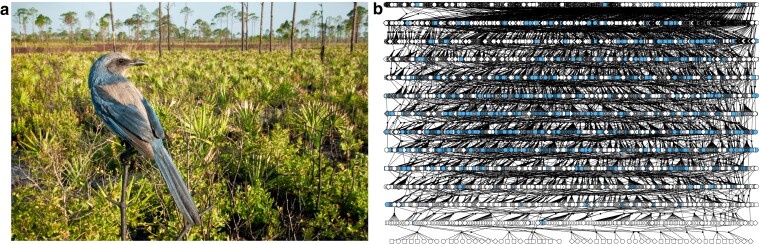
Image and population pedigree of the Florida Scrub-Jay (*A. coerulescens*). a) A banded Florida Scrub-Jay from the long-term demographic study at Archbold Biological Station. Photo courtesy of Reed Bowman. b) The population pedigree for Florida Scrub-Jays at Archbold Biological Station from 1969 to 2021. Light blue symbols indicate individuals who have been genotyped. *N* = 11,026.

The Florida Scrub-Jay (*Aphelocoma coerulescens*) is a federally Threatened, cooperatively breeding bird endemic to the US state of Florida (Fig. 1a) (Woolfenden and Fitzpatrick 1984). It is the sister taxon to all other Scrub-Jays (California Scrub-Jay, *Aphelocoma californica*; Island Scrub-Jay, *Aphelocoma insularis*; Woodhouse's Scrub-Jay, *Aphelocoma woodhouseii*; and Sumichrast's Scrub-Jay, *Aphelocoma sumichrasti*), which are all found west of the Mississippi River (McCormack *et al*. 2011; DeRaad *et al*. 2022). The estimated divergence time is ∼2.5 million years ago (McCormack *et al*. 2011). The Florida Scrub-Jay has been in decline due to anthropogenic development and fire suppression and currently exists in small, locally isolated populations across the state (Boughton and Bowman 2011). It is intensively monitored throughout its range with multiple well-characterized natural populations, including a long-term study at Archbold Biological Station in Venus, Florida. All individuals in this population have been uniquely banded and monitored since 1969, resulting in a 15-generation pedigree with near-complete fitness data for > 11,000 individuals (Fig. 1b) (Woolfenden and Fitzpatrick 1984; Fitzpatrick and Bowman 2016). This robust dataset has led to foundational knowledge in the behavior, demography, and life history of cooperative breeders (Woolfenden and Fitzpatrick 1984), with further work shedding light on social and environmental effects on lifetime fitness (Mumme *et al*. 2015; Fitzpatrick and Bowman 2016) and the causes and consequences of dispersal and immigration (Coulon *et al*. 2010; Aguillon *et al*. 2017; Suh *et al*. 2020, 2022; Summers *et al*. 2024). Studies of other populations of Florida Scrub-Jays have contributed to our understanding of the negative impacts of suburbanization (Thorington and Bowman 2003; Coulon *et al*. 2012), population dynamics (Breininger *et al*. 1999; Breininger and Carter 2003), ecological importance of periodic wildfire (Breininger *et al*. 2023), and the repercussions of translocations (Linderoth *et al*. 2023). Exhaustive genetic sampling of thousands of individuals at Archbold Biological Station has also allowed for the rare opportunity to study the evolution of small, natural populations, such as genetic population structure (Coulon *et al*. 2008, 2010), allele frequency changes (Chen *et al.* 2019; Driscoll *et al.* 2024), and the genetic consequences of inbreeding and reduced immigration (Chen *et al*. 2016; Nguyen *et al*. 2022), all of which are imperative to understand in the face of habitat fragmentation and the loss of genetic diversity in species worldwide (Thomas *et al*. 2004). To better characterize genetic variation present in the Florida Scrub-Jay, we must have a high-quality reference genome as a point of comparison.

A reference genome assembled using ALLPATHS-LG (Gnerre *et al*. 2011) for a male Florida Scrub-Jay was published as part of the Bird 10,000 Genomes Project (Feng *et al*. 2020) and then further improved and scaffolded with the aid of Hi–C reads (Driscoll *et al.* 2024). However, these previous assemblies were generated with Illumina short-read data, which may not have captured the full scope of genomic information, such as highly repetitive regions (Treangen and Salzberg 2012). Additionally, as birds have a ZW sex-determination system in which females are the heterogametic sex, these assemblies are missing the W chromosome. Here, we present a new chromosome-level genome assembly for a female Florida Scrub-Jay generated with long-read sequencing technology, chromosome conformation data, and a linkage map. This new long-read assembly is 1.33 Gb long, adds 270 Mb in length to the previous reference genomes, and contains 4 newly identified chromosomes to the Florida Scrub-Jay, including the W chromosome. We also provide annotations of repetitive and gene content, evaluations of the quality and contiguity of the data presented, and the first linkage map for this species.

## Materials and methods

### Sampling and genome sequencing

We collected fresh blood via venipuncture from an inbred, adult female Florida Scrub-Jay at Archbold Biological Station, Venus, Florida [approved by Cornell University Institutional Animal Care and Use Committee (IACUC 2010-0015), authorized by permit no. TE824723-8 issued by the US Fish and Wildlife Service, banding permit no. 07732 issued by the US Geological Survey, and permit no. LSSC-10-00205 issued by the Florida Fish and Wildlife Commission]. The University of Delaware DNA Sequencing & Genotyping Center extracted DNA from the blood sample using a high molecular weight extraction protocol, then prepared a Pacific Biosciences (PacBio) library and sequenced it on 3 SMRT Cells (Sequel IIe system).

Additionally, we performed 20 × coverage short-read whole-genome resequencing for 25 male and 25 female Florida Scrub-Jays, including the parents of the individual we sampled for PacBio sequencing, to aid in the identification of sex chromosomes based on differences in read depth between males and females. We extracted DNA from archived blood samples stored in Queen's lysis buffer using Qiagen DNeasy Blood and Tissue kits and sent DNA to Novogene (Sacramento, CA, USA) for PCR-free library preparation and 150 bp paired-end sequencing on an Illumina NovaSeq6000 platform.

### Linkage map generation

We created a linkage map using CRI-MAP v. 2.507 (Green *et al*. 1990) and the CRIGEN package (Liu and Grosz 2006). We used data from a previous study that genotyped 3,838 individuals at 12,210 SNPs using a custom Illumina iSelect Beadchip (Chen *et al*. 2016). We trimmed our pedigree to include only completely genotyped trios, then split it into 32 three-generation sub-families of ∼100 individuals each using the *crigen* function. Using a subset of 3,424 informative SNPs (1 SNP per scaffold of the short-read Florida Scrub-Jay genome assembly; Feng *et al.* 2020), we created a sparse (pre-framework) map. We calculated pairwise logarithm (base 10) of odds (LOD) scores using *twopoint* and assigned markers to linkage groups using the *autogroup* function. *Autogroup* uses an iterative process to assign markers to linkage groups with four levels of increasing stringency. The parameters we used for minimum LOD score, minimum number of informative meioses, maximum number of shared linkages, and minimum linkage ratio were: level 1 (100, 2.0, 2, 0.9), level 2 (50, 1.5, 3, 0.7), level 3 (10, 1.0, 5, 0.6), and level 4 (5, 0.4, 6, 0.5). We labelled linkage groups based on alignments with the Zebra Finch genome (NCBI accession GCA_000151805.2). Then, to construct each linkage group, we identified haplogroups using the *hap* function and ran *build* four times with different starting markers and a threshold of LOD > 5. We chose the longest map as the pre-framework map for each linkage group. To check marker order, we permuted up to five adjacent markers with the function *flips* to look for alternative marker orders with higher likelihood and iteratively updated the marker order until no better orders were found.

To expand the pre-framework map, we ran *twopoint* and *autogroup* on the full SNP set as above to assign the remaining markers to linkage groups. For each linkage group, we added markers onto the pre-framework map using *build* with a threshold of LOD > 5 and confirmed marker order with *flips*. When linkage groups had multiple equivalent best orders, we either removed markers with multiple potential orders or picked the order that was most consistent with the physical map. Finally, we used *fixed* to output the maximum likelihood recombination fractions and map distances for the sex-averaged map (setting SEX_EQ to 1) and the sex-specific map (setting SEX_EQ to 0). We used crimaptools v0.1 (https://github.com/susjoh/crimaptools, Johnston *et al*. 2016) to parse output files. To quantify sex differences in recombination rates (heterochiasmy), we calculated the deviation of the male-to-female genetic map length ratio from 1:1 (McAuley *et al*. 2024) and the heterochiasmy index, or the difference in the sex-specific genetic map lengths divided by the sex-averaged genetic map length (Malinovskaya *et al*. 2020).

### *De novo* genome assembly

To assemble the genome, we first used Cutadapt v. 2.3 (Martin 2011) to identify and discard PacBio HiFi raw reads with adapter sequences. Next, we created primary and alternate draft assemblies using Hifiasm v. 0.16.1 (Cheng *et al*. 2021) in HiFi-only mode with default parameters. We also ran Hifiasm in trio-binning mode, which leverages short-read data from the reference individual's parents to generate haplotype-resolved assemblies. We prepared the maternal and paternal reads by trimming adapters and filtering for quality with fastp v. 0.21.0 (Chen *et al*. 2018), merging paired-end reads with PEAR v. 0.9.11 (J. Zhang *et al*. 2014), and building a k-mer hash table for each set of reads using yak v. 0.1 (Li 2020). We compared the quality and contiguity of the four draft assemblies (primary, alternate, maternally resolved haplotype, and paternally resolved haplotype) with Quast v. 5.0.2 (Mikheenko *et al*. 2018) and Benchmarking Universal Single-Copy Orthologs (BUSCO) v. 5.2.2 (using the aves_odb10 and eukaryote_odb10 databases; Manni *et al.* 2021) and moved forward with the most contiguous assembly (the primary assembly). To further scaffold the genome, we used the 95.7 Gb of Hi–C reads generated by Dovetail Genomics for the short-read Florida Scrub-Jay genome assembly (Driscoll *et al.* 2024). We used the Arima Hi–C mapping pipeline (https://github.com/ArimaGenomics/mapping_pipeline) to map the paired-end Hi–C reads to the Hifiasm primary assembly and SALSA v. 2.3 (Ghurye *et al*. 2017) to join the contigs into scaffolds. We visualized the Hi–C contact map and manually curated the scaffolds to generate a chromosome-level genome assembly using the *juicer.sh*, *run-assembly-visualizer.sh*, and *run-asm-pipeline-post-review.sh* scripts from the Juicer v. 1.6 pipeline (Durand *et al*. 2016). To further improve contiguity, we ordered and oriented scaffolds given positional evidence from our linkage map using ALLMAPS v. 1.3.7 from the JCVI Utilities Library (Tang *et al*. 2015). For scaffolding, we used a linkage map with 4,477 SNPs that had additional markers added to three linkage groups (LG 34, 36, and Chr Z) using *build* and *flips* with a threshold of LOD > 3. If any marker locations in the linkage map conflicted with the ordering of contigs joined during the SALSA or Juicer scaffolding steps, we manually broke those scaffolds in disagreement and iteratively ran ALLMAPS until the genetic and physical positions for each linkage group were in concordance. We next screened the genome for organismal contaminants using the BlobToolKit suite v. 3.1.0 (Challis *et al*. 2020) with the *–busco*, *–hits*, and *–cov* flags. We also conducted a BLAST search (BLAST v. 2.10.0+; Camacho *et al.* 2009) against the publicly available Florida Scrub-Jay mitochondrial genome sequence (NCBI accession NC_051467.1) to identify and remove any mitochondrial contaminants in our assembly. We assigned scaffolds with sequence identity > 99% against the mitochondrial sequence and total length < 16.9 Kb (the length of the mitochondrial genome) as mitochondrial contaminants (DeRaad *et al*. 2023). Finally, we numbered the linkage groups according to homology with the Zebra Finch reference genome (*Taeniopygia guttata*; bTaeGut1.4.pri, NCBI accession GCA_003957565.4). Full descriptions of all software and options used for de novo genome assembly are available in Supplementary Table 1.

### Sex chromosome identification

We identified Z- and W-linked scaffolds using a two pronged approach: relative read depth and sequence homology to other bird species. Using whole-genome sequence data from 50 Florida Scrub-Jays, 25 males and 25 females, we followed the basic methodology from the findZX pipeline (Sigeman *et al*. 2022), a computational pipeline for sex chromosome identification. We processed each set of raw reads as follows: (1) trimmed adapter sequences and low-quality reads using Trim Galore (Krueger *et al*. 2023), (2) mapped the raw reads to the genome assembly using BWA-MEM (Li 2013), (3) filtered for read pairs that completely mapped in the expected orientation with a mapping quality greater than 20 using samtools *view* (Danecek *et al*. 2021), (4) marked and removed duplicate reads using sambamba (Tarasov *et al*. 2015), (5) filtered for reads with an edit distance of ≤ 2 using bamtools *filter* (Barnett *et al*. 2011), and (6) calculated per-basepair read depth and average read depth per scaffold across the genome, ignoring repetitive and low complexity regions, using a custom Bash script and samtools *mpileup* (Danecek *et al*. 2021). We then compared the average read depth of each scaffold between males and females in R using a series of *t*-tests with significance values Bonferroni-corrected for multiple comparisons. We putatively assigned scaffolds with significantly different average read depths as Z-linked if read depth was higher in males than in females and W-linked if read depth was higher in females than in males. We confirmed sex chromosome assignments by aligning each putatively sex-linked scaffold to its appropriate sex chromosome in the Zebra Finch reference genome and checking for sequence homology. As an additional check, we aligned W-linked scaffolds to the paternally-resolved haplotype assembly to confirm that they are missing from the male (ZZ) assembly. Finally, we labeled scaffolds as Z-linked or W-linked if they both yielded significant differences in read depth between males and females and displayed sequence homology to the Zebra Finch sex chromosomes. Full software parameters are available in Supplementary Table 1.

### Genome annotation

#### Repetitive element annotation

To annotate repetitive content across the genome, we first constructed a custom repeat library for the Florida Scrub-Jay using RepeatModeler v. 2.0.4 (Flynn *et al*. 2020) with the *-LTRStruct* flag. We then merged this library with a curated avian repeat library (Peona, Palacios-Gimenez, *et al*. 2021) and a curated repeat library of the closely related Steller's Jay (*Cyanocitta stelleri*; Benham *et al.* 2023). We used RepeatMasker v. 4.1.4 (Smit *et al*. 2013) to identify repetitive regions across the genome with the *-s* and *-xsmall* flags to implement a slow search and softmask the genome, respectively. To assess how sequencing technologies (*i.e.* short-read vs long-read) impacted repeat annotation, we compared the total counts and median lengths of transposable element (TE) superfamilies (Kapitonov and Jurka 2008) across both the previous Hi–C-scaffolded short-read assembly and the new long-read assembly using Wilcoxon rank sum tests.

#### Gene prediction and functional annotation

Robust and highly confident gene prediction leverages both RNA-seq and protein data. As such, we used the BRAKER pipeline v. 3.0.6, which integrates both types of data to train and execute the GeneMark-ETP and AUGUSTUS gene prediction tools (Stanke *et al*. 2006, 2008; Gotoh 2008; Iwata and Gotoh 2012; Buchfink *et al*. 2015; Hoff *et al*. 2016, 2019; Kovaka *et al*. 2019; Pertea and Pertea 2020; Brůna *et al*. 2021; Bruna *et al*. 2024). We obtained trimmed and filtered RNA-seq reads from Driscoll *et al.* (2024). These data consist of two lanes of 2 × 101 bp Illumina HiSeq sequencing generated from RNA extracted from liver, heart, kidney, and ovary tissues from one male (catalog number FMNH 396251) and one female (catalog number FMNH 396253) Florida Scrub-Jay (Peterson 1992; Driscoll *et al.* 2024). We used STAR v. 2.7.3 (Dobin *et al*. 2013) to align the RNA-seq reads to the softmasked genome assembly and Picard v. 2.27.4 (Broad Institute 2019) to assign read groups to each sample. We then executed BRAKER with the softmasked genome, aligned RNA-seq reads, and the *Vertebrata* protein sequence database from OrthoDB v.11 (Kuznetsov *et al*. 2023) as input data. We used InterProScan v. 5.69-101.0 (Jones *et al*. 2014) and a protein BLAST search against the Swiss-Prot database (The UniProt Consortium 2019) to assign functional annotations to the resulting gene set. Finally, we combined the outputs of BRAKER and InterProScan into a consensus gene annotation using the AGAT v. 1.2.0 suite of tools (Dainat 2023). Full descriptions of all software and options used for genome annotation are available in Supplementary Table 1.

### Genome completeness assessment

We used QUAST to calculate basic assembly quality statistics and BUSCO to assess expected gene content and completeness across the genome (as above). To explore synteny across the *Aves* group, we used minimap2 v. 2.26 (Li 2018) to generate whole genome–whole genome alignments of our assembly with publicly available genome assemblies for Zebra Finch, Chicken (*Gallus gallus*; GGswu, NCBI accession GCA_024206055.2), New Caledonian Crow (*Corvus moneduloides*; bCorMon1.pri, NCBI accession GCA_009650955.1), and the closely related California Scrub-Jay (*A. californica*; bAphCal1.0.hap1, NCBI accession GCA_028536675.1). We filtered for primary alignments with alignment lengths > 10 Kb and mapping quality > 40.

We compared our genome assembly and annotation quality with other publicly available avian genome assemblies and annotations. We downloaded genome assemblies, annotations, and protein sequence data from NCBI for the New Caledonian Crow, Hawaiian Crow (*Corvus hawaiiensis*; bCorHaw1.pri.cur, NCBI accession GCA_020740725.1), Hooded Crow (*Corvus cornix*; ASM73873v6, NCBI accession ASM73873v6), Collared Flycatcher (*Ficedula albicollis*; FicAlb1.5, NCBI accession GCA_000247815.2), and Zebra Finch. For each species, we calculated basic assembly quality statistics with QUAST and annotation statistics by inputting their publicly available annotation files (GFF) into the AGAT toolkit script *agat_sp_statistics.pl* (Dainat 2023). Finally, we explored the functions and similarities across the annotated gene sets by using OrthoVenn3 (Sun *et al*. 2023) to identify orthologous gene clusters.

## Results and discussion

### Linkage map

**Fig. 2.**
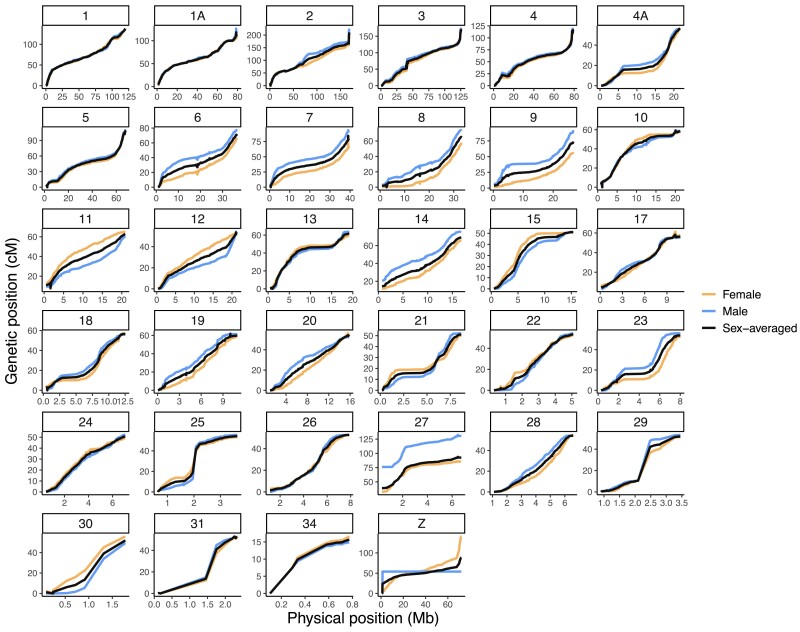
Sex-averaged and sex-specific Marey maps for the Florida Scrub-Jay, with genetic position (Kosambi cM) shown relative to physical location (Mb). See Supplementary Fig. 1 for a more detailed view of the linkage map and Supplementary Table 2 for the full linkage map.

Linkage map construction initially assigned 3,182 SNPs to 36 linkage groups in the pre-framework map. After expanding linkage analysis to the full SNP dataset, we assigned 12,151 SNPs to 41 linkage groups. We proceeded to build linkage maps for the 34 linkage groups that contained more than 5 markers (Fig. 2, Supplementary Fig. 1). Our framework map with marker order supported by LOD > 5 consists of 4,468 SNPs with a total sex-averaged autosomal genetic map length of 2446.78 cM and mean genetic distance between markers of 0.56 cM (± 1.33 cM). The female and male autosomal map lengths were 2373.56 and 2567.09 cM, respectively (Fig. 2, Supplementary Fig. 1). While we see higher recombination in males than females in the Florida Scrub-Jay, the magnitude of heterochiasmy in this species is low. The ratio of the male to female genetic map length deviated from 1:1 by −0.08 and the heterochiasmy index is 0.079. Previous studies of recombination rates in other avian species have found male:female genetic map length ratios that deviate from 1:1 by −0.22 to 0.36 (McAuley *et al*. 2024) and heterochiasmy index estimates ranging from −0.2 to 0.35 (Malinovskaya *et al*. 2020). We include the full linkage map in Supplementary Fig. 1 and Table 2.

### Genome sequencing and assembly

**Fig. 3.**
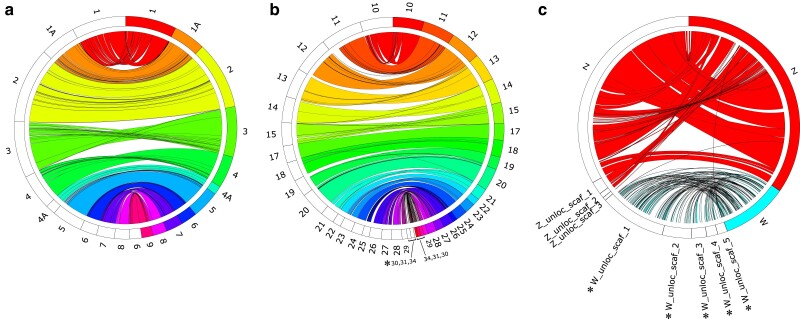
Sequence homology between the Florida Scrub-Jay and the Zebra Finch (*T. guttata*; bTaeGut1.4.pri) for a) macrochromosomes (1–9, 1A, 4A), b) microchromosomes (10–31, 34), and c) sex chromosomes (Z, W) and sex-linked scaffolds. The outer ring represents genome sequence divided into chromosomes/scaffolds: white bars on the left hemisphere represent Florida Scrub-Jay scaffolds and colored bars on the right hemisphere represent Zebra Finch scaffolds, with colored ribbons showing sequence alignment. Chromosomes and scaffolds labeled with an asterisk (*) are newly identified in the Florida Scrub-Jay. For clarity, we filtered the autosomes for alignments > 100 Kb and the sex chromosomes for alignments > 50 Kb. We created these plots with minimap v. 2.26 (Li 2018) and Circos v. 0.69-9 (Krzywinski *et al*. 2009) with code adapted from the online tutorial at: https://bioinf.cc/misc/2020/08/08/circos-ribbons.html.

PacBio HiFi long-read sequencing yielded 84 Gb of raw read data, with a mean read length of 14.55 Kb. We created four draft assemblies with Hifiasm: primary, alternate, maternally-resolved haplotype, and paternally-resolved haplotype. We moved forward with the primary assembly, as it was the most contiguous (LG50/NG50 of 17 contigs/20.98 Mb) and had the highest BUSCO scores of the four draft assemblies (97.1% completeness; Supplementary Table 3). Scaffolding with SALSA and Juicer generated an assembly with 699 scaffolds and an LG50/NG50 of 9 scaffolds/33.36 Mb (Supplementary Fig. 2, Table 1). Linkage map-aided scaffolding with ALLMAPS identified 34 linkage groups. Of these linkage groups, 31 (including the Z) were associated with complete chromosomes previously identified in the short-read Florida Scrub-Jay genome assembly based on homology with Zebra Finch (Driscoll *et al.* 2024). The remaining 3 linkage groups were newly assembled chromosomes with sequence homology to chromosomes 30, 31, and 34 in the Zebra Finch (Fig. 3). While the haploid chromosome number for the Florida Scrub-Jay is unknown because there is no karyotype data for this species, it is likely that some of the unplaced contigs in our assembly belong to additional microchromosomes. A typical avian karyotype has a 2*n* = 76–80 (Ellegren 2013), and the most closely-related species with cytogenetic data, the Blue Jay (*Cyanocitta cristata*), has a karyotype of 2*n* = 78 (Jovanović and Atkins 1969; Fernando *et al*. 2017).

**Table 1.** Basic assembly statistics for each step of the long-read Florida Scrub-Jay genome assembly.

	Hifiasm	+ SALSA2	+ Juicer Manual curation	+ ALLMAPS
Total length (bp)	1322553486	1322602486	1322601186	1330898477
Number of contigs/scaffolds	783	699	699	659
N50 (Mb)	17.71	33.36	33.36	68.05
L50	18	9	9	7
NG50 (Mb)	20.98	33.36	33.36	68.05
LG50	17	9	9	7
Longest contig/scaffold (Mb)	93.47	124.80	124.80	168.97
Number of *N*'s per 100 Kb	0.00	3.70	3.61	3.57
BUSCO scores (%) (*Aves*; *n* = 8338)	C: 97.1	C: 97.0	C: 97.2	C: 97.1
	S: 96.5	S: 96.4	S: 96.5	S: 96.4
	D: 0.6	D: 0.6	D: 0.7	D: 0.7
	F: 0.5	F: 0.5	F: 0.6	F: 0.6
	M: 2.4	M: 2.5	M: 2.2	M: 2.3
BUSCO scores (%) (*Eukaryota*; *n* = 255)	C: 98.8	C: 98.8	C: 98.8	C: 99.2
	S: 97.6	S: 98.0	S: 97.6	S: 98.0
	D: 1.2	D: 0.8	D: 1.2	D: 1.2
	F: 0.8	F: 0.8	F: 0.8	F: 0.4
	M: 0.4	M: 0.4	M: 0.4	M: 0.4

The Hifiasm column reports statistics for contigs, while all other columns report statistics for scaffolds. We calculated NG50/LG50 values using an estimated genome size of 1.3 Gb. The *Aves* row of BUSCO scores uses the aves_odb10 (2024-01-08) database with 8338 BUSCOs available. The *Eukaryota* row of BUSCO scores uses the eukaryote_odb10 (2024-01-08) database with 255 BUSCOs available. BUSCO parameters are as follows: C, complete; S, complete and single-copy; D, complete and duplicated; F, fragmented; M, missing (Manni *et al*. 2021).

Our whole genome assembly displayed high levels of synteny with other bird genomes, with 88% of the Florida Scrub-Jay genome placed in 35 chromosomes with 35, 34, 33, and 30 homologs in Zebra Finch, New Caledonian Crow, California Scrub-Jay, and Chicken, respectively (Supplementary Table 4). As expected, the whole-genome alignment with the most distantly related Chicken yielded the most rearrangements and sequence differences, while the alignment with the congeneric California Scrub-Jay yielded the fewest (Supplementary Fig. 3).

**Fig. 4.**
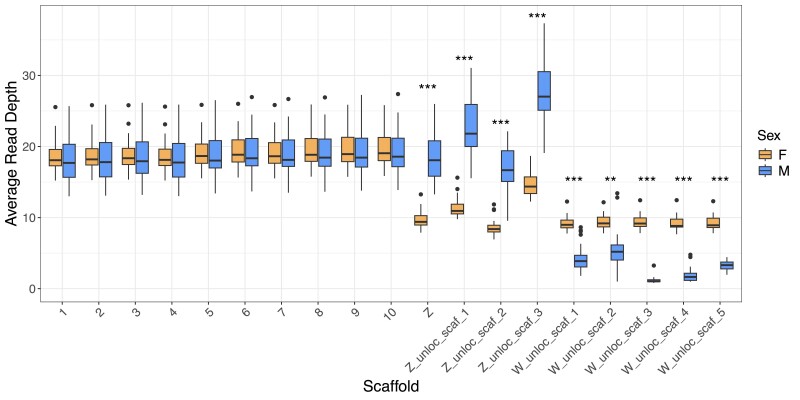
Average read depth of sex-linked scaffolds in 25 female (orange) and 25 male (blue) Florida Scrub-Jays. We include the first 10 autosomes for comparison. W-linked scaffolds show approximately twice the read depth in females when compared to males, while Z-linked scaffolds show approximately twice the read depth in males when compared to females. Scaffolds with significantly different average read depths from a *t*-test are indicated as follows: *P < 2.55 × 10^−5^, **P < 2.55 × 10^−6^, ***P < 2.55 × 10^−7^ (we used Bonferroni corrections for 392 comparisons).

Next, we identified sex-linked scaffolds by comparing average read depth per scaffold across 25 male and 25 female Florida Scrub-Jays. We found 18 scaffolds that significantly differed in depth between males and females. Due to their small size, we were only able to confirm 8 of these scaffolds, 3 Z-linked and 5 W-linked, as homologous to Zebra Finch sex chromosome sequence (Figs. 3c and 4). During this process, we discovered a single contig in the middle of our scaffolded Z chromosome that aligned to both Z and W sequence from multiple other bird species. We examined this contig in more detail using read depth in 1 kb sliding windows and discovered that it was a Z/W chimeric contig created during the first Hifiasm assembly step (Supplementary Fig. 4). We used a combination of evidence from sequence synteny with other bird species, read depth, examination of inter-chromosomal contacts using Hi–C reads in JuiceBox, and alignments with the paternally resolved haplotype Hifiasm assembly (Supplementary Fig. 4) to manually split this chimeric contig into a 4.7 Mb W-linked scaffold (W_unloc_scaf_3) and a 575 Kb Z-linked scaffold. We then inserted the Z-linked scaffold into the main Z chromosome using the linkage map-based scaffolder ALLMAPS (Fig. 3c).

The largest Z-linked scaffold, which corresponds to the Z linkage group, aligned to ∼99% of the Zebra Finch Z chromosome (Fig. 3c). The largest W-linked scaffold was equal in size to the entire Zebra Finch W chromosome (∼21 Mb), but the 5 additional W-linked scaffolds added 20.5 Mb in sequence, yielding a total of 41.5 Mb of sequence identified as part of the Florida Scrub-Jay W chromosome (Fig. 3c). Finally, we mapped all W-linked scaffolds onto the paternally-resolved haplotype assembly. After filtering using the scheme described previously, we yielded no alignments, further confirming the W assignment of these scaffolds. At the end of these analyses, we labeled the largest Z-linked scaffold as the Z chromosome, the 3 additional Z-linked scaffolds as unlocalized Z sequence, and the 5 W-linked scaffolds as unlocalized W sequence.

During decontamination screening with Blobtoolkit, one scaffold of length 57.8 Kb was identified as belonging to a non-Chordate (specifically, to *Drosophila melanogaster* in Arthropoda; Supplementary Fig. 5). We believe this result is a computational artifact because the BLAST alignments had low identity scores (*pident* = 76–77%) and together represented a very small percentage of the total scaffold length (1,964 bp of 57.8 Kb = ∼3.3% of total scaffold length). We therefore concluded this scaffold belonged to the Florida Scrub-Jay and retained it in the final genome assembly. Our assembly does not include the mitochondrial genome: we found no significant BLAST hits to the Florida Scrub-Jay mitochondria in our final assembly or in our raw PacBio HiFi reads.

Our final genome assembly is 1.33 Gb long and consists of 659 scaffolds, 87.6% of which belonged to 33 named autosomes and the sex chromosomes, with an LG50/NG50 of 7 scaffolds/68.05 Mb and a BUSCO completeness score of 97.1% with respect to the aves_odb10 database (Table 1). These quality measures are similar to those of other published genome assemblies in Corvidae and Passeriformes (Table 2). The Florida Scrub-Jay has the longest genome amongst the species considered, and is comparable in length to the more closely-related California Scrub-Jay (1.35 Gb; DeRaad *et al.* 2023).

**Table 2.** Summary statistics for the Florida Scrub-Jay (*A. coerulescens*) genome and annotation compared to other similar bird species in Corvidae and Passeriformes with annotated genomes (New Caledonian Crow, *C. moneduloides*; Hawaiian Crow, *C. hawaiiensis*; Hooded Crow, *C. cornix*; Collared Flycatcher, *Ficedula albicollis*; Zebra Finch, *Taeniopygia gutatta*).

	Florida Scrub-Jay	New Caledonian Crow	Hawaiian Crow	Hooded Crow	Collared Flycatcher	Zebra Finch
NCBI accession	GCA_041296385.1	GCA_009650955.1	GCA_020740725.1	GCA_000738735.6	GCA_000247815.2	GCA_003957565.4
Annotation date and method	2024, BRAKER3	2020, Gnomon	2022, Gnomon	2014, BLAST and Scipio	2016, Gnomon	2021, Gnomon
Genome assembly length (Gb)	1.3	1.1	1.2	1.0	1.1	1.1
Genome assembly N50 (Mb)	68.05	74.7	76.3	73.7	64.7	71
No. of genes	17,964	16,167	16,414	14,435	15,400	16,520
Mean gene length (bp)	22,762	36,436	35,242	37,961	33,382	33,996
Number of CDS	26,689	43,047	43,147	36,899	26,464	41,214
Mean length of CDS (bp)	1,912	2,293	2,332	2,275	1,941	2,282
No. of exons	313,816	638,785	648,529	555,077	336,665	607,674
Mean exon length (bp)	162	291	283	293	241	299
Mean no. exons per gene	11.8	14.1	14.3	14.3	12.1	14.0
No. of introns	287,127	563,402	573,808	490,050	294,478	536,658
Genome BUSCO scores (%) (*Aves*; *n* = 8338)	C: 97.1	C: 96.8	C: 97.3	C: 94.8	C: 96.6	C: 96.4
	S: 96.4	S: 96.3	S: 96.8	S: 94.4	S: 96.1	S: 95.9
	D: 0.7	D: 0.5	D: 0.5	D: 0.4	D: 0.5	D: 0.5
	F: 0.6	F: 0.5	F: 0.5	F: 0.6	F: 0.7	F: 0.7
	M: 2.3	M: 2.7	M: 2.2	M: 4.6	M: 2.7	M: 2.9

Note that genome assembly length, genome assembly N50, and genome BUSCO scores are genome summary statistics, while all other statistics are gene annotation summary statistics. BUSCO parameters are as follows: C, complete; S, complete and single-copy; D, complete and duplicated; F, fragmented; M, missing (Manni *et al*. 2021).

### Genome annotation

#### Repetitive content

**Fig. 5.**
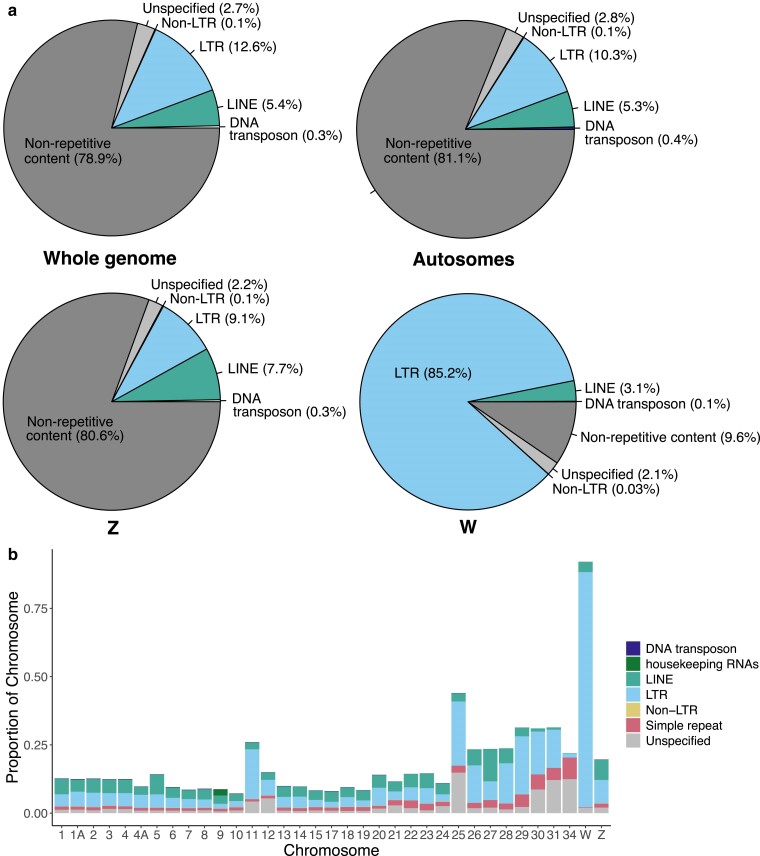
Repeat annotation of the Florida Scrub-Jay genome. a) Summary of interspersed repeat content across the whole-genome, autosomes, the Z chromosome and Z-linked scaffolds, and the W chromosome and W-linked scaffolds. b) Repetitive content of each chromosome, colored by TE superfamily (Kapitonov and Jurka 2008).

We identified 281 Mb of interspersed repeats throughout the genome, comprising 21.13% of the total genome length (Fig. 5a). Repetitive content of the new long-read genome was more than double that of the previous short-read assemblies, which both had an estimated interspersed repeat content of 8.7% (Supplementary Table 5). The new long-read assembly added ∼189 Mb of identified repetitive content and yielded more DNA transposons, housekeeping RNAs, long interspersed nuclear elements (LINEs), long tandem repeats (LTRs), non-LTR retroelements (*e.g.* short interspersed nuclear elements), satellite sequences, simple repeats, and unclassified repetitive elements (Supplementary Fig. 6a). This increase in repetitive content between our long-read and short-read genome assemblies matches similar patterns observed in sparrows (Benham *et al*. 2024). Avian genomes have long been thought to have low repeat content (< 10%; Ellegren 2010; Zhang *et al.* 2014b), but genomes assembled with new long-read sequencing technologies are indicating that repeat content in bird genomes has previously been underestimated.

The median length of all TE superfamilies, with the exception of Non-LTRs, was longer in the long-read assembly when compared to the short-read assembly (Supplementary Fig. 6b), highlighting the effectiveness of long-read sequencing technology in assembling both shorter and longer repetitive elements. The most common element across the genome was LTR retrotransposons (12.6%) followed by LINEs (5.4%) (Fig. 5), aligning with patterns seen in other avian genomes, particularly amongst songbirds (Kapusta and Suh 2017; Boman *et al*. 2019; Weissensteiner *et al*. 2020). The W-linked scaffold was a strong outlier in repetitive content, with ∼90% of the sequence characterized as interspersed repeats spanning the entire chromosome (Fig. 5a, Supplementary Fig. 7). The majority of these elements were classified as LTRs, supporting the role of the W chromosome as a haven for some repeat families in birds (Peona, Blom, *et al*. 2021; Peona, Palacios-Gimenez, *et al*. 2021).

#### Gene content

We annotated a total number of 17,964 genes throughout the genome, with a mean gene length of 22.8 Kb and a mean of 11.8 exons per gene (Table 2). Of the identified genes, 92.5% were annotated with functional information and 84.8% had an associated Gene Ontology (GO) term. BUSCO completeness of the transcriptome was 95.4% for the *Aves* database and 98.4% for the *Eukaryota* database. Mean gene length, mean number exons per gene, and all other annotation quality measures are comparable to that of other similar avian species (Table 2). We annotated the greatest number of genes (17,964) amongst the species considered (Table 2). Using OrthoVenn3 (Sun *et al*. 2023) to visualize overlaps in identified proteins between the 6 species, we found 21,191 clusters in 217,460 proteins, 11,579 of which were shared between all species, and 3,972 of which were single-copy clusters (Supplementary Fig. 8). The 3 largest clusters shared between all species were assigned GO terms of “synaptic membrane adhesion”, “vesical targeting”, and “visual perception”.

### Conclusion

We report a high-quality genome assembly, associated annotation, and a linkage map for the Florida Scrub-Jay. Using a combination of long-read sequencing, Hi–C data, and our linkage map, we generated a highly contiguous genome assembly, with a size of 1.33 Gb, an NG50 of 68 Mb, and a BUSCO completeness score of 97.1% (Table 1). Additionally, we provide the first assembly of the W chromosome in this species, as well as three newly identified chromosomes. This annotated genome assembly and linkage map will facilitate more detailed genomic analyses, such as the exploration of haplotype dynamics across space and time and the genetic architecture of fitness, and open the door for new and exciting questions about the biology, ecology, and evolution of this Federally Threatened species.

## Supplementary Material

jkae232_Supplementary_Data

## Data Availability

The genome assembly is available on NCBI under the accession GCA_041296385.1. Raw sequence reads are available on NCBI under BioProjects PRJNA1076903 and PRJNA1097984. The genome annotation, repeat library and repeat annotation, and linkage maps are available in Figshare at: figshare.com/projects/Florida_Scrub-Jay_genome_assembly/220939 under the following DOIs: https://doi.org/10.6084/m9.figshare.27037915.v1, https://doi.org/10.6084/m9.figshare.27037921.v1, https://doi.org/10.6084/m9.figshare.27037966.v1, https://doi.org/10.6084/m9.figshare.27037945.v1, https://doi.org/10.6084/m9.figshare.27037942.v1. All additional data and associated code are available at: github.com/faye-romero/FSJ-genome. Supplemental material available at G3 online.
