# Supplementary material for: A new high-quality genome assembly and annotation for the threatened Florida Scrub-Jay (Aphelocoma coerulescens)
Source: G3 (Bethesda). 2024 Sep 27;14(12):jkae232. doi: 10.1093/g3journal/jkae232 (PMC11631490; doi:10.1093/g3journal/jkae232)
Supplement: jkae232_Supplementary_Data [file jkae232_supplementary_data.zip › Figure_S1_G3-2024-405021.docx]

**Figure S1.** A genetic linkage map for the Florida Scrub-Jay. Each column represents a linkage group (chromosome). Black tick marks represent marker locations of the LOD5 framework map, with linkage group lengths measured in Kosambi cM (y axis). We created this plot with the R package LinkageMapView (Ouellette et al. 2018).
