# Supplementary material for: A new high-quality genome assembly and annotation for the threatened Florida Scrub-Jay (Aphelocoma coerulescens)
Source: G3 (Bethesda). 2024 Sep 27;14(12):jkae232. doi: 10.1093/g3journal/jkae232 (PMC11631490; doi:10.1093/g3journal/jkae232)
Supplement: jkae232_Supplementary_Data [file jkae232_supplementary_data.zip › Figure_S2_G3-2024-405021.docx]

**
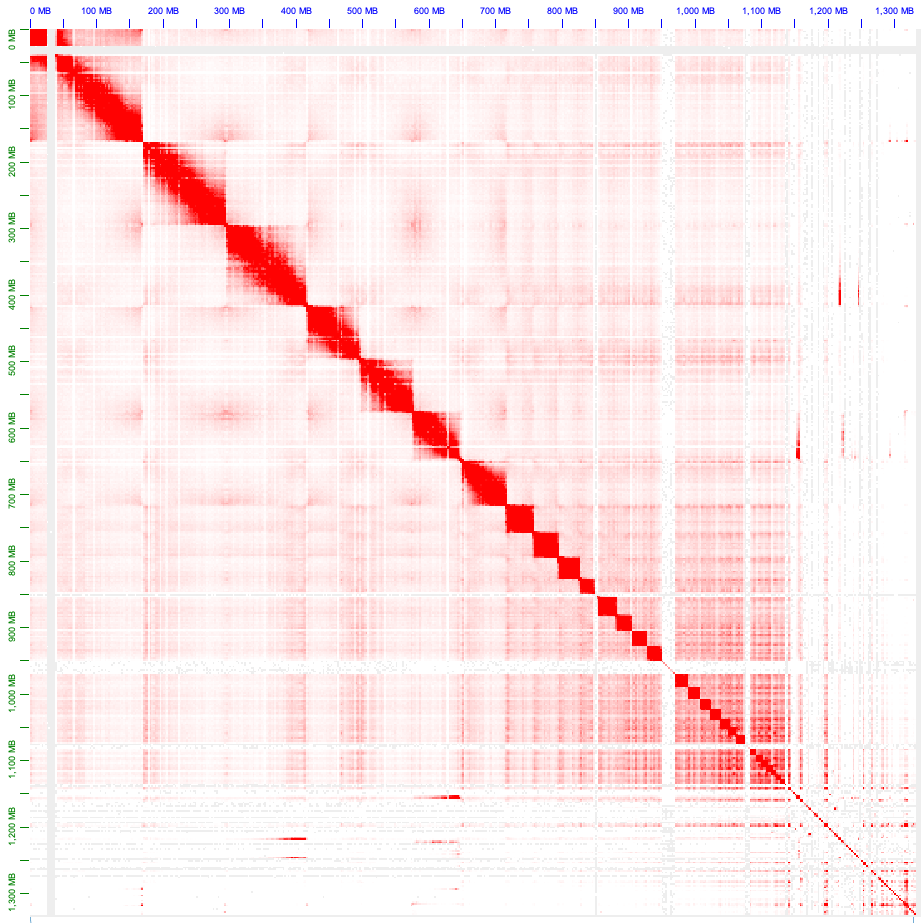
**

**Figure S2.** Hi-C contact map of the final genome assembly. We created this map using the Juicer/JuiceBox suite (Durand et al. 2016).
