# Supplementary material for: A new high-quality genome assembly and annotation for the threatened Florida Scrub-Jay (Aphelocoma coerulescens)
Source: G3 (Bethesda). 2024 Sep 27;14(12):jkae232. doi: 10.1093/g3journal/jkae232 (PMC11631490; doi:10.1093/g3journal/jkae232)
Supplement: jkae232_Supplementary_Data [file jkae232_supplementary_data.zip › Figure_S3_G3-2024-405021.docx]

**
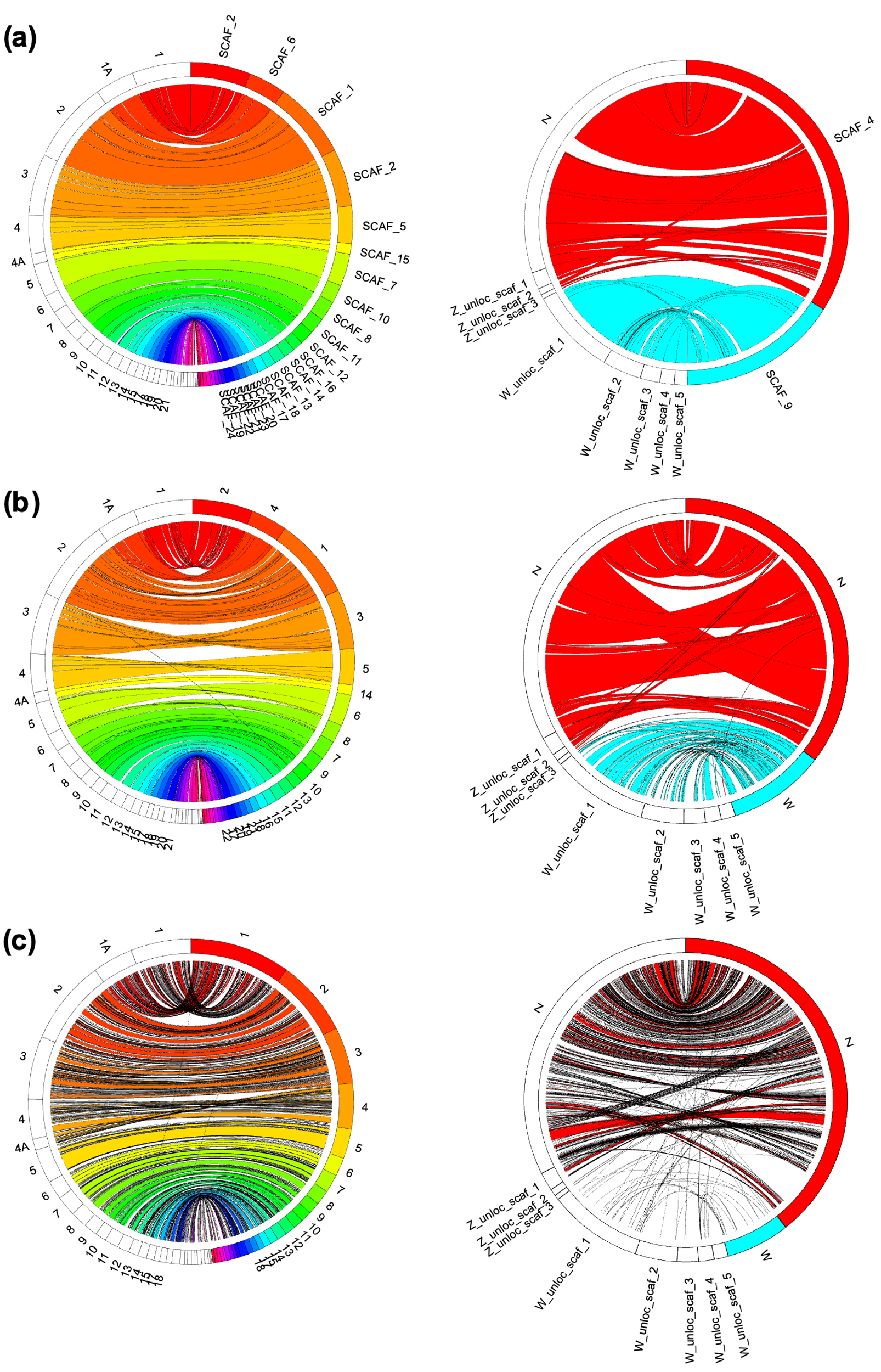
**

**Figure S3.** Sequence homology between the new long-read Florida Scrub-Jay genome assembly and the **(a)** California Scrub-Jay, **(b)** New Caledonian Crow, and **(c)** Chicken. We display autosomal alignments on the left and sex chromosome alignments on the right. The outer ring of each plot represents genome sequence divided into chromosomes/scaffolds: white bars on the left hemisphere represent Florida Scrub-Jay scaffolds and colored bars on the right hemisphere represent the reference species sequence, with colored ribbons showing sequence alignment. The California Scrub-Jay reference genome did not have named chromosomes; we therefore sorted the genome by synteny with Florida Scrub-Jay chromosomes. For clarity, we filtered the plots using the following filtering schemes: Chicken autosomes, alignment length (*alen*) > 50 kbp; Chicken sex chromosomes, *alen* > 5 kbp; New Caledonian Crow autosomes, *alen* > 100 kbp; New Caledonian Crow sex chromosomes, *alen* > 100 kbp; California Scrub-Jay autosomes, *alen* > 800 kbp; California Scrub-Jay sex chromosomes, *alen* > 400 kbp. We also removed overlapping chromosome labels for clarity; for the full list of syntenic chromosome pairings, see Table S4. We created these plots with minimap2 v. 2.26 (Li 2018) and Circos v. 0.69-9 (Krzywinski et al. 2009) with code adapted from the online tutorial https://bioinf.cc/misc/2020/08/08/circos-ribbons.html.
