# Supplementary material for: A new high-quality genome assembly and annotation for the threatened Florida Scrub-Jay (Aphelocoma coerulescens)
Source: G3 (Bethesda). 2024 Sep 27;14(12):jkae232. doi: 10.1093/g3journal/jkae232 (PMC11631490; doi:10.1093/g3journal/jkae232)
Supplement: jkae232_Supplementary_Data [file jkae232_supplementary_data.zip › Figure_S4_G3-2024-405021.docx]

**Figure S4.** Z chromosome/W chromosome chimeric contig discovery and splitting process. **(a)** Circos plot of sequence alignments (colored ribbons) between the original incorrectly-assembled Florida Scrub-Jay sex chromosomes (white left hemisphere) and Zebra Finch sex chromosomes (colored right hemisphere) shows that a section of the Florida Scrub-Jay Z aligns to Zebra Finch W. For clarity, we filtered for alignment lengths > 50 Kb. We created this plot with minimap2 v. 2.26 (Li 2018) and Circos v. 0.69-9 (Krzywinski et al. 2009) with code adapted from the online tutorial https://bioinf.cc/misc/2020/08/08/circos-ribbons.html. **(b)** Average read depth across the original incorrectly-assembled Florida Scrub-Jay Z chromosome in 25 female (orange) and 25 male (blue) individuals in 10 Kb windows confirms that region has nearly 0 read depth in males. **(c)** Average read depth across the chimeric contig in 25 female (orange) and 25 male (blue) individuals in 1 Kb windows suggests the first 4.7 Mb is W-linked while the last 575 Kb is Z-linked. **(d)** Sequence alignment between a single contig from the paternally-resolved haplotype Hifiasm assembly (x-axis) and the chimeric contig (y-axis). The first 4.7 Mb of the chimeric contig yielded no alignment, while the last 575 Kb of the chimeric contig aligned with unbroken, 100% alignment identity to the paternally-resolved haplotype assembly, strongly suggesting a W-linked and a Z-linked region, respectively. Warmer colors indicate higher percent alignment identity. We created this plot with Mummer v. 4.0 (Marçais et al. 2018). **(e)** Hi-C contact maps of the chimeric contig before (left) and after (right) we manually split the contig into two: a 4.7 Mb W-linked contig and 575 Kb Z-linked contig. Blue boxes represent contig boundaries and red indicates chromosomal contacts. As our Hi-C reads were generated from a male individual and mapped to our female genome assembly, we expect to see chromosomal contacts in Z-linked regions but not in W-linked regions. We created these maps using the Juicer/JuiceBox suite (Durand et al. 2016).
