# Supplementary material for: A new high-quality genome assembly and annotation for the threatened Florida Scrub-Jay (Aphelocoma coerulescens)
Source: G3 (Bethesda). 2024 Sep 27;14(12):jkae232. doi: 10.1093/g3journal/jkae232 (PMC11631490; doi:10.1093/g3journal/jkae232)
Supplement: jkae232_Supplementary_Data [file jkae232_supplementary_data.zip › Figure_S5_G3-2024-405021.docx]

**Figure S5.** Decontamination screening results. **(a)** Snail plot describing summary statistics of the long-read genome assembly. **(b)** Plot displaying cumulative length of sequences assigned to different taxonomic categories. **(c)** Blob plot of base coverage across the genome (y-axis) against proportion of GC content across the genome (x-axis). Histograms show the distribution of sequence lengths along each axis. We viewed and created all plots with the BlobToolKit pipeline (Challis et al. 2020).
