# Supplementary material for: A new high-quality genome assembly and annotation for the threatened Florida Scrub-Jay (Aphelocoma coerulescens)
Source: G3 (Bethesda). 2024 Sep 27;14(12):jkae232. doi: 10.1093/g3journal/jkae232 (PMC11631490; doi:10.1093/g3journal/jkae232)
Supplement: jkae232_Supplementary_Data [file jkae232_supplementary_data.zip › Figure_S6_G3-2024-405021.docx]

**Figure S6.** Comparison of repetitive element annotation between the Florida Scrub-Jay Illumina short-read assembly scaffolded with Hi-C data (Driscoll and Beaudry *et al.* 2024) and the new PacBio HiFi long-read genome assembly. (a) Number of repetitive elements annotated across each genome, separated by transposable element (TE) superfamily (Kapitonov and Jurka 2008). TE counts for each superfamily differed significantly between the short-read and long-read assemblies (Proportion test; p < 0.001 for all). (b) Median length of TEs annotated across each genome, separated by TE superfamily (Kapitonov and Jurka 2008). Median TE length for each superfamily differed significantly between the short-read and long-read assemblies (Wilcoxon rank sum test; p < 0.001 for all except Non-LTRs, which was non-significant). Significance codes are as follows: * = p < 0.05, ** = p < 0.01, *** = p < 0.001.
