# Supplementary material for: A new high-quality genome assembly and annotation for the threatened Florida Scrub-Jay (Aphelocoma coerulescens)
Source: G3 (Bethesda). 2024 Sep 27;14(12):jkae232. doi: 10.1093/g3journal/jkae232 (PMC11631490; doi:10.1093/g3journal/jkae232)
Supplement: jkae232_Supplementary_Data [file jkae232_supplementary_data.zip › Figure_S7_G3-2024-405021.docx]

**Figure S7.** Transposable element composition across 500 Kb windows for each chromosome. Note that the y-axis range varies across chromosomes. We grouped transposable elements by superfamily (Kapitonov and Jurka 2008).
