# Supplementary material for: A new high-quality genome assembly and annotation for the threatened Florida Scrub-Jay (Aphelocoma coerulescens)
Source: G3 (Bethesda). 2024 Sep 27;14(12):jkae232. doi: 10.1093/g3journal/jkae232 (PMC11631490; doi:10.1093/g3journal/jkae232)
Supplement: jkae232_Supplementary_Data [file jkae232_supplementary_data.zip › Figure_S8_G3-2024-405021.docx]

**Figure S8.** Venn diagram displaying the number of orthologous gene clusters across 6 species: Florida Scrub-Jay, *Aphelocoma coerulescens*; Hawaiian Crow, *Corvus hawaiiensis*; Hooded Crow, *Corvus cornix*; Collared Flycatcher, *Ficedula albicollis*; Zebra Finch, *Taeniopygia gutatta*, and New Caledonian Crow, *Corvus moneduloides*. The vertical bar chart displays the number of proteins identified for each species. The horizontal bar chart displays the number of orthologous genes shared across 6 species, 5 species, etc. We created these figures with OrthoVenn3 (Sun et al. 2023).
