# Supplementary material for: A new high-quality genome assembly and annotation for the threatened Florida Scrub-Jay (Aphelocoma coerulescens)
Source: G3 (Bethesda). 2024 Sep 27;14(12):jkae232. doi: 10.1093/g3journal/jkae232 (PMC11631490; doi:10.1093/g3journal/jkae232)
Supplement: jkae232_Supplementary_Data [file jkae232_supplementary_data.zip › Supplemental_Material_References_G3-2024-405021.docx]

**REFERENCES**

Challis R, Richards E, Rajan J, Cochrane G, Blaxter M. 2020. BlobToolKit – Interactive Quality Assessment of Genome Assemblies. G3 Genes|Genomes|Genetics. 10(4):1361–1374. doi:10.1534/g3.119.400908.

Driscoll RMH, Beaudry FEG, Cosgrove EJ, Bowman R, Fitzpatrick JW, Schoech SJ, Chen N. 2024. Allele frequency dynamics under sex-biased demography and sex-specific inheritance in a pedigreed jay population. Genetics. 227(3):iyae075. doi:10.1093/genetics/iyae075.

Durand NC, Shamim MS, Machol I, Rao SSP, Huntley MH, Lander ES, Aiden EL. 2016. Juicer Provides a One-Click System for Analyzing Loop-Resolution Hi-C Experiments. Cell Syst. 3(1):95–98. doi:10.1016/j.cels.2016.07.002.

Feng S, Stiller J, Deng Y, Armstrong J, Fang Q, Reeve AH, Xie D, Chen G, Guo C, Faircloth BC, et al. 2020. Dense sampling of bird diversity increases power of comparative genomics. Nature. 587(7833):252–257. doi:10.1038/s41586-020-2873-9.

Green P, Evans I, Maddox J. 1990. CRI-MAP: Improved. Available from: https://www.animalgenome.org/tools/share/crimap/.

Kapitonov VV, Jurka J. 2008. A universal classification of eukaryotic transposable elements implemented in Repbase. Nat Rev Genet. 9(5):411–412. doi:10.1038/nrg2165-c1.

Krzywinski MI, Schein JE, Birol I, Connors J, Gascoyne R, Horsman D, Jones SJ, Marra MA. 2009 Jun 18. Circos: An information aesthetic for comparative genomics. Genome Res. doi:10.1101/gr.092759.109. [accessed 2024 Mar 13]. https://genome.cshlp.org/content/early/2009/06/15/gr.092759.109.

Li H. 2018. Minimap2: pairwise alignment for nucleotide sequences. Bioinformatics. 34(18):3094–3100. doi:10.1093/bioinformatics/bty191.

Manni M, Berkeley MR, Seppey M, Simão FA, Zdobnov EM. 2021. BUSCO Update: Novel and Streamlined Workflows along with Broader and Deeper Phylogenetic Coverage for Scoring of Eukaryotic, Prokaryotic, and Viral Genomes. Molecular Biology and Evolution. 38(10):4647–4654. doi:10.1093/molbev/msab199.

Marçais G, Delcher AL, Phillippy AM, Coston R, Salzberg SL, Zimin A. 2018. MUMmer4: A fast and versatile genome alignment system. PLOS Computational Biology. 14(1):e1005944. doi:10.1371/journal.pcbi.1005944.

Ouellette LA, Reid RW, Blanchard SG, Brouwer CR. 2018. LinkageMapView—rendering high-resolution linkage and QTL maps. Bioinformatics. 34(2):306–307. doi:10.1093/bioinformatics/btx576.

Sun J, Lu F, Luo Y, Bie L, Xu L, Wang Y. 2023. OrthoVenn3: an integrated platform for exploring and visualizing orthologous data across genomes. Nucleic Acids Research. 51(W1):W397–W403. doi:10.1093/nar/gkad313.
