# Supplementary material for: A new high-quality genome assembly and annotation for the threatened Florida Scrub-Jay (Aphelocoma coerulescens)
Source: G3 (Bethesda). 2024 Sep 27;14(12):jkae232. doi: 10.1093/g3journal/jkae232 (PMC11631490; doi:10.1093/g3journal/jkae232)
Supplement: jkae232_Supplementary_Data [file jkae232_supplementary_data.zip › Table_S1_G3-2024-405021.docx]

| **Purpose** | **Software** | **Version** | **Options** |
| --- | --- | --- | --- |
| *De novo genome assembly* | | | |
| Remove adapter sequences from PacBio HiFi raw reads | Cutadapt | 2.3 | --discard-trimmed --overlap=35 -b ATCTCTCTCAACAACAACAACGGA  GGAGGAGGAAAAGAGAGAGAT -b ATCTCTCTCTTTTCCTCCTCCTCCG  TTGTTGTTGTTGAGAGAGAT |
| Genome assembly | Hifiasm (HiFi-only assembly mode) | 0.16.1 | default |
| Trim and filter parental short reads | fastp | 0.21.0 | default |
| Merge parental paired-end short reads | PEAR | 0.9.11 | default |
| Build k-mer hash tables for parental short reads | yak | 0.1 (r56)  Commit: 76db8e8 | count -k31 -b37 -t16  count -k31 -b37 -t16 |
| Genome assembly | Hifiasm (Trio-binning assembly mode) | 0.16.1 | default |
| Map Hi-C reads to draft genome assembly | Arima Genomics Mapping Pipeline | February 8, 2019 release (Document Part Number A160156 v01) | default |
| Scaffold assembly | SALSA | 2.3 | -e GATC -i 5 -m yes |
| Scaffold and visualize assembly | Juicer | 1.6 | *juicer.sh*  -s DpnII -C 180000000 --assembly  *run-assembly-visualizer.sh*  default  *run-asm-pipeline-post-review.sh*  --sort-output -r |
| Scaffold assembly | ALLMAPS (JCVI Utilities Libraries) | 1.3.7 | -m jcvi.assembly.allmaps merge  -m jcvi.assembly.allmaps path |
| Decontamination screening | BlobToolKit | 3.1.0 | add --busco  add --hits --taxrule bestsumorder  add --cov |
| Decontamination screening | BLAST | 2.10.0+ | -outfmt 6 |
| Whole genome alignments | Minimap2 | 2.26 | -x asm20 |
| Plot whole genome alignments | Circos | 0.69-9 | default |
| *Sex chromosome identification* | | | |
| Trim and filter Illumina raw reads | Trim Galore | 0.6.10 | --paired --fastqc |
| Map raw reads to genome assembly | BWA-MEM | 0.7.4 | default |
| Filter reads for quality | Samtools | 1.9 | view -b -f 0x2 -F 260 -q 20 |
| Mark and remove duplicate reads | Sambamba | 1.0.1 | markdups --remove-duplicates |
| Filter reads for quality | Bamtools |  | filter -tag NM:<=2 |
| Calculate read depth | Samtools | 1.9 | mpileup --positions --fasta-ref |
| Sequence alignment | MUMmer | 4.0 | nucmer -t 8  delta-filter -i 20 -l 1000  mummerplot –color --png |
| *Genome annotation* | | | |
| Annotate *de novo* repeat families | RepeatModeler | 2.0.4 | -LTRStruct |
| Identify and classify repetitive elements | RepeatMasker | 4.1.4 | -gff -s -a -xsmall |
| Align RNA-seq reads to genome assembly | STAR | 2.7.3 | --twopassMode Basic --readFilesCommand zcat --outSAMstrandField intronMotif |
| Assign read groups to RNA-seq reads | Picard | 2.27.4 | AddOrReplaceReadGroups -VALIDATION_STRINGENCY LENIENT -RGPL RNA |
| Gene annotation | BRAKER3 | 3.0.6 | --prot_seq --rnaseq_sets_ids --gff3 |
| Assign functional information to gene annotation | InterProScan | 5.69-101.0 | --goterms --iprlookup |
| Gene annotation | BLAST | 2.10.0+ | blastp -evalue 0.000001 -outfmt 6 |
| Merge gene annotations, calculate summary statistics | AGAT | 1.2.0 | *agat_sp_manage_functional_annotation.pl*  --gff3 --blast --db --interpro --pcds  *agat_sp_statistics.pl*  --gff --gs 1330898477 -d  *agat_sp_functional_statistics.pl*  --gff --gs 1330898477 |

**Table S1.** Descriptions of programs and software used during genome assembly and annotation. Any options listed are in addition to default parameters.
