# Supplementary material for: A new high-quality genome assembly and annotation for the threatened Florida Scrub-Jay (Aphelocoma coerulescens)
Source: G3 (Bethesda). 2024 Sep 27;14(12):jkae232. doi: 10.1093/g3journal/jkae232 (PMC11631490; doi:10.1093/g3journal/jkae232)
Supplement: jkae232_Supplementary_Data [file jkae232_supplementary_data.zip › Table_S3_G3-2024-405021.docx]

|  | **Primary (HiFi-only mode)** | **Alternate (HiFi-only mode)** | **Maternally-resolved haplotype (Trio-binning mode)** | **Paternally-resolved haplotype (Trio-binning mode)** |
| --- | --- | --- | --- | --- |
| **Total length (bp)** | 1322553486 | 984436143 | 1293735984 | 738092893 |
| **Number of contigs/scaffolds** | 783 | 4851 | 990 | 1133 |
| **N50 (Mb)** | 17.71 | 0.95 | 13.39 | 5.35 |
| **L50** | 18 | 280 | 25 | 34 |
| **NG50 (Mb)** | 20.98 | 0.59 | 13.39 | 0.41 |
| **LG50** | 17 | 487 | 25 | 215 |
| **Longest contig/scaffold (Mb)** | 93.47 | 6.53 | 81.63 | 34.17 |
| **Number of N’s per 100 kbp** | 0.00 | 0.00 | 0.00 | 0.00 |
| **BUSCO scores (*Aves* database)** | C: 97.1%  S: 96.5%  D: 0.6%  F: 0.5%  M: 2.4% | C: 76.1%  S: 75.1%  D: 1.0%  F: 0.9%  M: 23.0% | C: 92.8%  S: 87.7%  D: 5.1%  F: 0.5%  M: 6.7% | C: 49.4%  S: 48.3%  D: 1.1%  F: 0.5%  M: 50.1% |

**Table S3.** Assembly statistics for each draft assembly created with Hifiasm v. 0.16.1. We calculated NG50/LG50 values using an estimated genome size of 1.3 Gb. BUSCO parameters are as follows: C: Complete, S: Complete and single-copy, D: Complete and duplicated, F: Fragmented, M: Missing (Manni et al. 2021).
