# Supplementary material for: A new high-quality genome assembly and annotation for the threatened Florida Scrub-Jay (Aphelocoma coerulescens)
Source: G3 (Bethesda). 2024 Sep 27;14(12):jkae232. doi: 10.1093/g3journal/jkae232 (PMC11631490; doi:10.1093/g3journal/jkae232)
Supplement: jkae232_Supplementary_Data [file jkae232_supplementary_data.zip › Table_S4_G3-2024-405021.docx]

| **Florida Scrub-Jay** | **Zebra Finch** | **California Scrub-Jay** | **New Caledonian Crow** | **Chicken** |
| --- | --- | --- | --- | --- |
| 1 | 1 | SCAF_2 | 2 | 1 |
| 1A | 1A | SCAF_6 | 4 | 1 |
| 2 | 2 | SCAF_1 | 1 | 2 |
| 3 | 3 | SCAF_3 | 3 | 3 |
| 4 | 4 | SCAF_5 | 5 | 4 |
| 4A | 4A | SCAF_15 | 14 | 4 |
| 5 | 5 | SCAF_7 | 6 | 5 |
| 6 | 6 | SCAF_10 | 8 | 6 |
| 7 | 7 | SCAF_8 | 7 | 7 |
| 8 | 8 | SCAF_11 | 9 | 8 |
| 9 | 9 | SCAF_12 | 10 | 9 |
| 10 | 10 | SCAF_16 | 13 | 10 |
| 11 | 11 | SCAF_14 | 12 | 11 |
| 12 | 12 | SCAF_13 | 11 | 12 |
| 13 | 13 | SCAF_18 | 15 | 13 |
| 14 | 14 | SCAF_17 | 16 | 14 |
| 15 | 15 | SCAF_20 | 18 | 15 |
| 17 | 17 | SCAF_23 | 21 | 17 |
| 18 | 18 | SCAF_21 | 19 | 18 |
| 19 | 19 | SCAF_22 | 20 | 19 |
| 20 | 20 | SCAF_19 | 17 | 20 |
| 21 | 21 | SCAF_24 | 22 | 21 |
| 22 | 22 | SCAF_30 | 27 | 22 |
| 23 | 23 | SCAF_26 | 23 | 23 |
| 24 | 24 | SCAF_28 | 25 | 24 |
| 25 | 25 | SCAF_33 | 29 | 25 |
| 26 | 26 | SCAF_35 | 24 | 26 |
| 27 | 27 | SCAF_27 | 26 | 27 |
| 28 | 28 | SCAF_29 | 28 | 28 |
| 29 | 29 | SCAF_31 | 30 | 33 |
| 30 | 30 | SCAF_35 | 32 | NA |
| 31 | 31 | NA | 34 | NA |
| 34 | 34 | NA | NA | NA |

**Table S4.** Syntenic autosomes between the Florida Scrub-Jay, Zebra Finch, California Scrub-Jay, New Caledonian Crow, and Chicken based on sequence homology. Cells with an “NA” indicate that we did not find a conclusive homolog to the Florida Scrub-Jay chromosome in question. See Figures 3 and S3 for visual sequence alignments.
