# Supplementary material for: A new high-quality genome assembly and annotation for the threatened Florida Scrub-Jay (Aphelocoma coerulescens)
Source: G3 (Bethesda). 2024 Sep 27;14(12):jkae232. doi: 10.1093/g3journal/jkae232 (PMC11631490; doi:10.1093/g3journal/jkae232)
Supplement: jkae232_Supplementary_Data [file jkae232_supplementary_data.zip › Table_S5_G3-2024-405021.docx]

|  | **Version 1 (Illumina)** | **Version 2 (Illumina + Hi-C)** | **New assembly (PacBio + HiC)** |
| --- | --- | --- | --- |
| *Specimen information* | | | |
| **Sample ID** | 1713-13619 | 1713-49378 | 1873-10779 |
| **Sex** | Male | Male | Female |
| **Year of sampling** | 2010 | 2018 | 2021 |
| **Birth year** | 2010 | 2012 | 2019 |
| *Assembly statistics* | | | |
| **Total length (bp)** | 1060889110 | 1060969718 | 1330898477 |
| **Number of contigs/scaffolds** | 1678 | 878 | 659 |
| **N50 (Mb)** | 7.66 | 74.35 | 68.05 |
| **L50** | 40 | 5 | 7 |
| **NG50 (Mb)** | 5.45 | 64.04 | 68.05 |
| **LG50** | 58 | 7 | 7 |
| **Longest contig/scaffold (Mb)** | 29.17 | 156.03 | 168.97 |
| **Number of N’s per 100 kbp** | 1907.36 | 1914.34 | 3.57 |
| **BUSCO scores (%) (*Aves* database)** | C: 97.1  S: 96.7  D: 0.4  F: 0.5  M: 2.4 | C: 97.2  S: 96.8  D: 0.4  F: 0.5  M: 2.3 | C: 97.1  S: 96.4  D: 0.7  F: 0.6  M: 2.3 |
| *Repetitive element annotation statistics (% of genome)* | | | |
| **Total interspersed repeats** | 8.72 | 8.76 | 21.13 |
| **Retroelements** | 7.69 | 7.74 | 18.07 |
| **LTR elements** | 3.16 | 3.21 | 12.55 |
| **DNA transposons** | 0.34 | 0.34 | 0.34 |
| **Satellites** | 0.09 | 0.07 | 4.20 |
| **Unclassified** | 0.7 | 0.68 | 2.72 |

**Table S5.** Assembly and repetitive element annotation statistics for all three Florida Scrub-Jay genome assemblies: a short-read assembly from a male (Feng et al. 2020), the short-read assembly scaffolded with Hi-C data (Driscoll and Beaudry *et al.* 2024), and the new long-read assembly from a female (presented here). Specimen information for the Hi-C scaffolded genome refers to the individual used to generate Hi-C data. We calculated NG50/LG50 values using an estimated genome size of 1.3 Gb. BUSCO parameters are as follows: C: Complete, S: Complete and single-copy, D: Complete and duplicated, F: Fragmented, M: Missing (Manni et al. 2021).
